# Job resources do not mitigate the impact of job demands for workers with depression: Patricia Ots

**DOI:** 10.1093/eurpub/ckac130.214

**Published:** 2022-10-25

**Authors:** P Ots, AC Keller, E Altrock, SKR van Zon, S Brouwer

**Affiliations:** 1 Department of Health Sciences, University Medical Center Groningen, Groningen, Netherlands; 2 Department of Psychology, University of Groningen, Groningen, Netherlands

## Abstract

**Introduction:**

Jobs characterized by low to moderate job demands and high job resources are associated with better work outcomes among healthy workers, yet it remains unclear whether this is the case for workers with depression. This study examined whether depression moderates the relationship between job demands, job resources, and maintaining employment.

**Methods:**

Data from the longitudinal population-based Lifelines cohort study were matched with register data on employment status from Statistics Netherlands (n = 55,950). The two-way interaction between job demands and depression and the three-way interaction between job demands, job resources and depression were examined in a zero-inflated Poisson regression model with path 1 including a binary employment outcome and path 2 a count variable including months out of employment.

**Results:**

The interaction effect of job demands and depression on being employed was significant (b=-0.22, 95% CI: -0.44; 0.01), showing that workers without depression were more likely to be employed whereas workers with depression were less likely to be employed if they had high job demands. The three-way interaction between job demands, job resources, and depression was significant for months out of employment (b = 0.15, 95% CI: 0.01; 0.29), indicating that workers with depression had more months out of employment when reporting high job demands and high job resources compared to workers without depression.

**Discussion:**

Although increasing resources to prevent negative work outcomes may be beneficial for workers without depression, this approach might be limited for the vulnerable subgroup of workers with depression.

**Key messages:**

- While job demands are associated with a higher likelihood of employment during follow-up for workers without depression, this is not the case for workers with depression.
- Job resources do not help workers with depression who face high job demands.

